# AI applications in musculoskeletal imaging: a narrative review

**DOI:** 10.1186/s41747-024-00422-8

**Published:** 2024-02-15

**Authors:** Salvatore Gitto, Francesca Serpi, Domenico Albano, Giovanni Risoleo, Stefano Fusco, Carmelo Messina, Luca Maria Sconfienza

**Affiliations:** 1 Department of Biomedical Sciences for Health, Università degli Studi di Milano, Via Cristina Belgioioso 173, Milan, 20157 Italy; 2 IRCCS Istituto Ortopedico Galeazzi, Milan, Italy; 3 Dipartimento di Scienze Biomediche, Chirurgiche ed Odontoiatriche, Università degli Studi di Milano, Milan, Italy; 4 Scuola di Specializzazione in Radiodiagnostica, Università degli Studi di Milano, Milan, Italy

**Keywords:** Artificial intelligence, Bone neoplasms, Fractures (bone), Musculoskeletal diseases, Osteoarthritis

## Abstract

This narrative review focuses on clinical applications of artificial intelligence (AI) in musculoskeletal imaging. A range of musculoskeletal disorders are discussed using a clinical-based approach, including trauma, bone age estimation, osteoarthritis, bone and soft-tissue tumors, and orthopedic implant-related pathology. Several AI algorithms have been applied to fracture detection and classification, which are potentially helpful tools for radiologists and clinicians. In bone age assessment, AI methods have been applied to assist radiologists by automatizing workflow, thus reducing workload and inter-observer variability. AI may potentially aid radiologists in identifying and grading abnormal findings of osteoarthritis as well as predicting the onset or progression of this disease. Either alone or combined with radiomics, AI algorithms may potentially improve diagnosis and outcome prediction of bone and soft-tissue tumors. Finally, information regarding appropriate positioning of orthopedic implants and related complications may be obtained using AI algorithms. In conclusion, rather than replacing radiologists, the use of AI should instead help them to optimize workflow, augment diagnostic performance, and keep up with ever-increasing workload.

**Relevance statement** This narrative review provides an overview of AI applications in musculoskeletal imaging. As the number of AI technologies continues to increase, it will be crucial for radiologists to play a role in their selection and application as well as to fully understand their potential value in clinical practice.

**Key points**

• AI may potentially assist musculoskeletal radiologists in several interpretative tasks.

• AI applications to trauma, age estimation, osteoarthritis, tumors, and orthopedic implants are discussed.

• AI should help radiologists to optimize workflow and augment diagnostic performance.

**Graphical Abstract:**

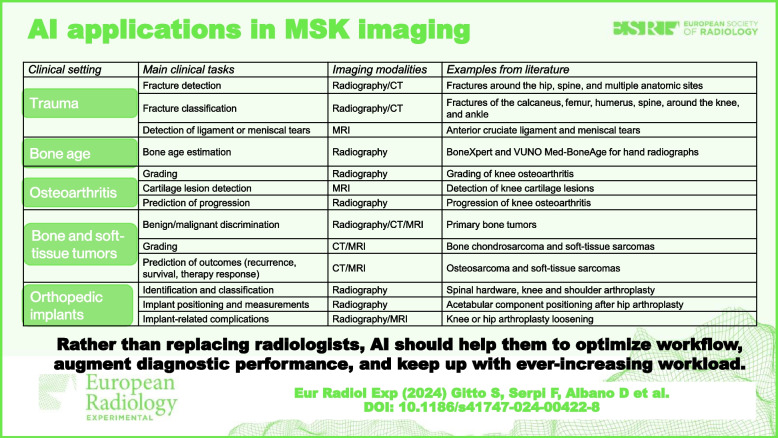

## Introduction

The term “artificial intelligence” (AI) refers to a field of computer science focused on allowing computers to mimic human cognitive functions [1]. This includes machine learning, which is a domain of AI enabling computers to learn and detect patterns in data without being explicitly programmed [2]. In turn, deep learning is a domain of machine learning (and therefore of AI) which can perform superior classification tasks [3]. In musculoskeletal imaging, AI, machine learning, and deep learning may assist radiologists in every step of the workflow, including both interpretative—such as detection and characterization of abnormalities—and non-interpretative tasks. The latter group includes (but is not limited to) the management of radiologic requests [4], protocoling [5], and production of images [6], similarly to what happens in any other imaging subspecialty. As they are not specific for musculoskeletal imaging, a detailed discussion on non-interpretative tasks is beyond the scope of this review. Thus, this narrative review mainly focuses on interpretative tasks and provides the reader with an up-to-date overview of AI applications in musculoskeletal imaging. A range of musculoskeletal disorders are discussed using a clinical-based approach, including those most often addressed in AI literature, such as trauma, bone age estimation, osteoarthritis, tumors, and orthopedic implants. A general overview of the clinical tasks achieved through AI in these fields of musculoskeletal imaging is offered in Table 1.

**Table 1:** Overview of the clinical tasks achieved through artificial intelligence (AI) systems in five fields of musculoskeletal imaging

*Clinical setting*	*Main clinical tasks*	*Imaging modalities*	*Examples from literature*
Trauma	Fracture detection	Radiography/CT	Fractures around the hip [7], spine [8], multiple anatomic sites [9]
	Fracture classification	Radiography/CT	Fractures of the calcaneus [10], femur [11], humerus [12], spine [13], around the knee [14], and ankle [15]
	Detection of ligament or meniscal tears	MRI	Anterior cruciate ligament and meniscal tears [16]
Bone age	Bone age estimation	Radiography	BoneXpert [17] and VUNO Med-BoneAge [18] for hand radiographs
Osteoarthritis	Grading	Radiography	Grading of knee osteoarthritis [19]
	Cartilage lesion detection	MRI	Detection of knee cartilage lesions [20]
	Prediction of progression	Radiography	Progression of knee osteoarthritis [21]
Bone and soft-tissue tumors	Benign/malignant discrimination	Radiography/CT/MRI	Primary bone tumors [22]
	Grading	CT/MRI	Bone chondrosarcoma [23–25] and soft-tissue sarcomas [26]
	Prediction of outcomes (recurrence, survival, therapy response)	CT/MRI	Osteosarcoma [27–29] and soft-tissue sarcomas [30]
Orthopedic implants	Identification and classification	Radiography	Spinal hardware [31], knee [32] and shoulder [33] arthroplasty
	Implant positioning and measurements	Radiography	Acetabular component positioning after hip arthroplasty [34]
	Implant-related complications	Radiography/MRI	Knee or hip arthroplasty loosening [35]

## Musculoskeletal trauma

Trauma is one of the most common reasons for patients presenting to emergency department. It represents a high cost for health care systems and missed or delayed diagnosis may lead to increased mortality and morbidity [36]. With the increasing growth of imaging utilization in the emergency setting, radiologists are constantly under pressure. It is estimated that there is approximately 4% error rate in imaging interpretation by a trained radiologist [37]. The risk of misinterpretation is higher when radiological exams are interpreted by non-radiology clinicians [38], which often occurs at night, when a consulting radiologist is not always available in every hospital. AI has the potential to reduce workload and improve diagnosis in the emergency settings [36].

AI has been applied to different imaging modalities, such as radiography, computed tomography (CT), and magnetic resonance imaging (MRI), with a special focus on radiography. To improve case collection and development of algorithms, deidentified radiography datasets have been created, for example MURA (musculoskeletal radiographs). MURA includes close to 41,000 radiographs of the upper extremity, which are all labeled as “normal” or “abnormal” by expert radiologists [39]. Several AI algorithms have been applied to fracture detection and classification. Lindsey et al. developed a deep neural network to detect and localize fractures on radiographs, which resulted in an improved diagnostic accuracy in fracture identification by emergency medicine clinicians with the assistance of AI [40]. Krogue et al. developed a deep learning-based hip fracture detection and classification model, which improved residents’ performance approximating that of unaided fellowship-trained attendings [41]. Gale et al. developed a DenseNet based architecture to detect hip fractures from frontal pelvic radiographs, which achieved equivalent performance compared to a human radiologist [7]. Chen et al. developed a ResNeXt architecture which was transferred to abdominal radiographs to identify vertebral fractures, with only slightly inferior performance compared to physicians such as radiologists and orthopedic surgeons (average accuracy of 76.8% for the physicians *versus* accuracy of 73% for the model) [8]. Thus, this model could be useful to assist physicians in the identification of vertebral fractures as incidental findings. Overall, in the mentioned studies, radiography-based algorithms had comparable performance to trained musculoskeletal radiologists. Thus, healthcare professionals without radiology training or residents in training may benefit the most in fracture detection, especially in centers with limited access to specialized musculoskeletal radiologists.

**Fig. 1:**
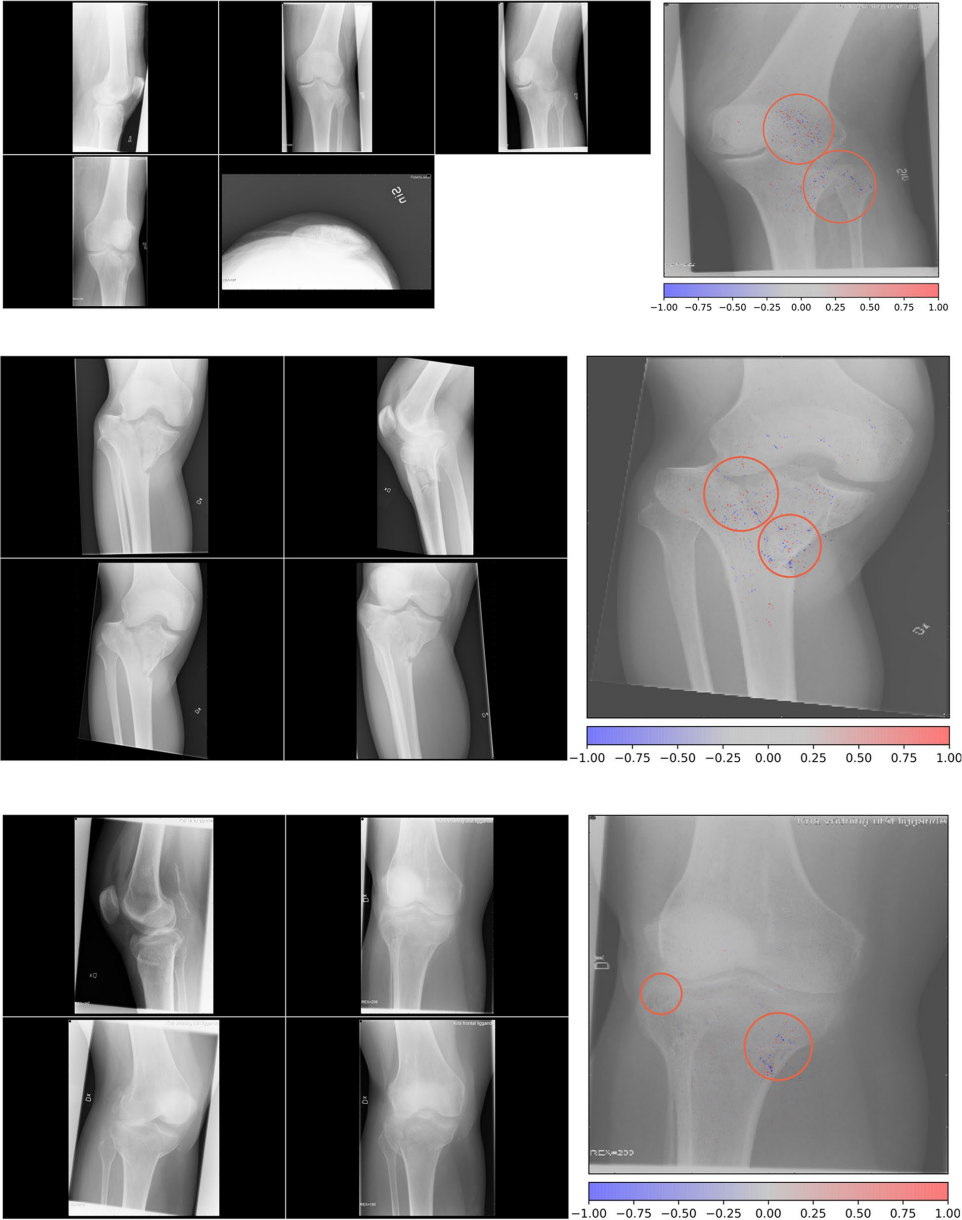
Example of fracture detection and classification method based on neural networks. Image areas where the network focuses on are shown as colored dots. Colored dots seem to cluster close to fracture lines, suggesting that the network appropriately finds these areas to contain relevant information. Adapted from: Lind A et al. [14] [open-access article distributed under the terms of the Creative Commons Attribution License (CC BY)]

Most of these algorithms are specific to a single anatomic area or body part. However, to be useful in practice, they will need to be combined in one interconnected software module that is capable to detect a fracture in any anatomic region. In a multicenter study, Jones et al. used 715,343 radiographs across 16 anatomic regions and an ensemble of 10 convolutional neural networks for fracture detection, with a mean area under the curve (AUC) above 0.98 in half of the anatomic sites [9]. Ma et al. developed a two-step approach to first detect the anatomic region among 20 different bones and thereafter to classify fractures, with an accuracy of 90% [42]. Regarding fracture classification, Chung et al. developed a proximal humerus fracture classification model based on Neer criteria using antero-posterior radiographs [12]. Tanzi et al. and Lind et al. developed deep learning models to classify proximal femur fractures [11] and fractures around the knee [14], respectively, based on the AO-OTA (Arbeitsgemeinschaft für Osteosynthesefragen-Orthopaedic Trauma Association) classification system. Olczak et al. trained a deep learning model to classify ankle fractures over a dataset of 4,941 patients achieving an average AUC of 0.90 [15]. Li et al. developed a deep learning model to classify vertebral fractures based on the Genant classification using plain lateral radiographs from 941 patients and achieved an AUC of 0.919−0.99 [13]. An example of fracture detection/classification method based on deep learning is shown in Fig. 1.

In CT, Jin et al. developed a deep learning model to identify rib fractures with a sensitivity of 92.9% [43]. Zhou et al. developed a convolutional neural network model which combined clinical information with CT to detect and classify rib fractures into recent, healing, and old fractures [44]. Pranata et al. built a model to identify and precisely localize calcaneal fractures [45]. Farda et al. built a convolutional neural network model to classify calcaneal fractures into the four Sanders categories with 72% accuracy [10]. Other models based on CT images focused on classification of osteoporotic vertebral compression and femoral neck fractures [46, 47].

Regarding MRI, Bien et al. showed a deep learning model which could perform multiple functions, such as detection of anterior cruciate ligament and meniscal tears, with however lower sensitivity for anterior cruciate ligament tears and lower specificity for meniscal tears compared to radiologists, respectively [16]. Liu et al. developed a deep learning model to improve sensitivity for anterior cruciate ligament tear detection [48], without significant difference compared to radiologists. Liu et al. developed a deep learning-based cartilage lesion model, with a high diagnostic performance and good intra-observer agreement for detecting cartilage degeneration and acute cartilage injury [20]. Kim et al. developed a deep learning algorithm to detect rotator cuff tear with 87% accuracy [49]. Hong et al. developed an AI model to analyze the efficacy of knee ligament trauma repair [50].

Ultrasound-based AI algorithms have been only mildly investigated compared to other imaging modalities. AI in ultrasound is mainly linked to classification, segmentation and diagnosis [51].

Beyond pure imaging interpretation, AI offers other applications in musculoskeletal trauma that can impact the management of patients. For example, it helps to predict the probability for occult posterior malleolar fracture in patients with known tibial shaft fracture [52] or to identify patients with tibial shaft fracture at risk for infection after operative treatment [53]. Despite the great potential of AI, further studies are needed to implement the use of AI in clinical practice, considering it as a diagnostic support for radiologists and clinicians and not a substitute of them.

## Bone age estimation

Correct assessment of bone age is important for different clinical fields, such as pediatric endocrinology, orthodontics, and orthopedics, as well as for legal medicine issues [54]. Bone age assessment is currently based on two main different methods: the Greulich-Pyle and the Tanner-Whitehouse methods, both focused on the analysis of the epiphyses and diaphysis morphology on hands radiographs. The Greulich-Pyle method is an atlas that contains reference images from hand radiographs, which were collected from 1931 to 1942 from upper-middle class Caucasian children in Ohio, USA [54, 55]. Bone age attribution is made by comparing a child hand radiographs with reference images in the atlas. The Tanner-Whitehouse method is based on data collected between 1950 and 1960 from children of average socioeconomic class in the UK, with further update in 2001 [56]. It evaluates maturity scores from the radius, ulna, carpals, and 13 hand short bones. Some of these bones are evaluated and categorized into stages ranging from A to I; then, a total score is calculated and converted into bone age [54, 56]. However, these conventional methods are time-consuming and prone to intra- and inter-observer variability.

Bone age assessments have become a major target of the machine learning community. The task is a typical object detection and classification problem of deep learning, with promising results [57]. Most of the studies are focused on left hand and wrist radiographs, with very few papers dealing with MRI, CT, and ultrasound. Radiographs in fact are faster, and radiation exposure is relatively low and considered safe [58]. In the history of bone age assessment, the first radiography-based automated techniques were introduced without the support of AI algorithms, such as HANDX system [59] and computer-based skeletal aging scoring system (CASAS) [60] introduced in 1989 and 1994, respectively. More recently, with the advancements of AI, many studies were conducted on AI-based bone age assessment solutions. BoneXpert (2008) is an automatic AI system which is widely used in Europe [17]. It uses feature extraction techniques and calculates bone age by analyzing the left-hand radiograph based on 13 bones, with improvement of time efficiency in daily clinical practice. Similarly, VUNO Med-BoneAge [18] and HH-boneage.io solution [61] are respectively semi-automatic and fully automatic AI systems to assess bone age on hand radiographs. MediAI-BA solution analyzes seven epiphysis-metaphysis growth regions [62].

Although different AI methods exist, the assessment of bone age in different ethnicities still represent a limit in most cases. In fact, different populations show different rates of skeletal maturation [63]. Therefore, most AI bone age assessment methods, particularly those based on Greulich-Pyle method, might be inaccurate with population of different ethnicity [57]. In addition, patients with congenital or acquired bone abnormalities or with previous surgery still necessitate manual assessment.

In conclusion, AI in bone age assessment is a useful tool that can assist radiologists, reducing workload and inter- and intra-observer variability. AI-assisted interpretation of bone age can also improve accuracy among junior readers [64]. However, multi-center and multi-national clinical trials are warranted to overcome the limitations of currently available AI methods.

## Osteoarthritis

AI research studies have been mainly focused on classification and prediction tasks in osteoarthritis [65]. Automated classification tasks may be highly beneficial to perform quantitative or semiquantitative analysis, which are essential to clinical routine but time-consuming for radiologists and subject to interobserver variability. However, the main challenge lies in creating individualized prediction models for osteoarthritis progression or development. Particularly, treatment plans may be targeted to the needs of individual patients prior to irreversible morphologic joint degeneration, including lifestyle changes (such as weight loss) at a timeframe during which disease progression may still be reversible [65].

These prediction models are multifactorial, and AI may help combining clinical risk factors for osteoarthritis with imaging biomarkers. The workflow for building AI models includes several steps. First, the clinical problem must be defined, including the definition of predictors (such as imaging biomarkers and clinical risk factors) and outcomes. Second, data are extracted and prepared for AI analysis, including dataset partition into training and test cohorts for model tuning and testing, respectively, as well as dimensionality reduction and class balancing in the training cohort to reduce the number of predictors and compensate for unbalanced datasets, respectively. Third, model training and hyperparameter optimization are performed to obtain AI models which predict the associations between predictor variables and outcomes. Finally, model performance is evaluated on the test cohort, which can be either internal if a single dataset is split into training and test cohorts or independent if two separate datasets are available for analysis. The latter approach is preferred as it improves the generalizability of the model [65].

In research studies dealing with osteoarthritis, AI-based classifications and predictions have been performed using deep learning, conventional machine learning, or ensemble machine learning approaches. Deep learning has been employed to analyze imaging data not only for binary classifications but also for quantitative or semiquantitative grading. Particularly, radiograph-based convolutional neural networks were used to automatically determine knee osteoarthritis grade with the Kellgren-Lawrence system, achieving an AUC of 0.93 [19]. On MRI, an automated method for cartilage lesion detection using convolutional neural networks showed sensitivity and specificity of 84% and 85%, respectively [20]. Conventional machine learning models have been built upon preidentified imaging data and demographics to predict future development or progression of osteoarthritis. For instance, radiographic and pain progression of knee osteoarthritis could be predicted with high accuracy (AUC of 0.86 and 0.95, respectively) when clustering subjects based on radiographic and pain progressive abnormalities and then using clinical variables to build machine learning models for predicting the probabilities of belonging to each cluster [66]. Finally, ensemble models incorporating both deep learning and conventional machine learning approaches based on imaging data have been employed for prediction purposes. Particularly, convolutional neural networks were used to evaluate the probability of knee osteoarthritis progression according to the Kellgren-Lawrence grade on radiographs [21]. In the same study, prognosis estimation was improved by combining deep learning prediction with clinical information using a gradient boosting machine, resulting in an AUC of 0.79 [21].

In conclusion, AI may potentially aid radiologists in identifying and grading abnormal findings of osteoarthritis more quickly and efficiently, also resulting in higher reproducibility. Additionally, by integrating clinical and imaging data, AI may help radiologists to predict the onset of osteoarthritis and its progression, thus enabling the implementation of preventive treatment strategies at early stages of the disease and resulting in decreased disability [65].

## Bone and soft-tissue tumors

Computer-aided diagnosis of bone tumors has been of interest for more than 50 years [67]. The first studies described a probabilistic approach based on patients’ demographics and imaging findings [67]. Recently, research studies have shifted away from radiologists entering imaging findings into computers, and towards direct presentation of medical images to AI models. The use of AI has been investigated for several applications in musculoskeletal tumor imaging, including primary or metastatic bone and soft-tissue lesions, although it is still at research stage [68]. Particularly, while skeletal metastases are relatively common, bone and soft-tissue sarcomas or primary malignant tumors are rare and highly heterogeneous, thus representing a challenge for AI model development.

Unsurprisingly, a recent systematic review focused on AI applied to musculoskeletal oncology showed that machine learning papers rapidly increased over the years [68]. Conventional machine learning and deep learning accounted for 77% and 23% of the included studies, respectively [68]. The bulk of conventional machine learning papers was related to radiomics applied to CT and MRI [68]. Nowadays, attention is focused on AI and radiomics as emerging tools to noninvasively provide information regarding diagnosis and outcome [69, 70].

Radiomics refers to the extraction and analysis of quantitative features from medical images, known as radiomic features, which may be used to support decision-making algorithms [71]. In musculoskeletal oncology, most AI-based radiomic studies focused on prediction of diagnosis—such as benign *versus* malignant tumor discrimination [72] or tumor grading [73]—and outcome—such as therapy response [27, 74], recurrence [28, 75], and survival [29]. In particular, several diagnosis-related studies dealt with benign *versus* malignant (or intermediate, like atypical cartilaginous or lipomatous tumors) discrimination and grading in skeletal cartilaginous tumors [23–25, 76], lipomatous soft-tissue tumors [77, 78] and soft-tissue sarcomas [26]. Most outcome-related studies dealt with therapy response, recurrence, or survival prediction in osteosarcoma [27–29] and soft-tissue sarcomas [30].

**Fig. 2:**
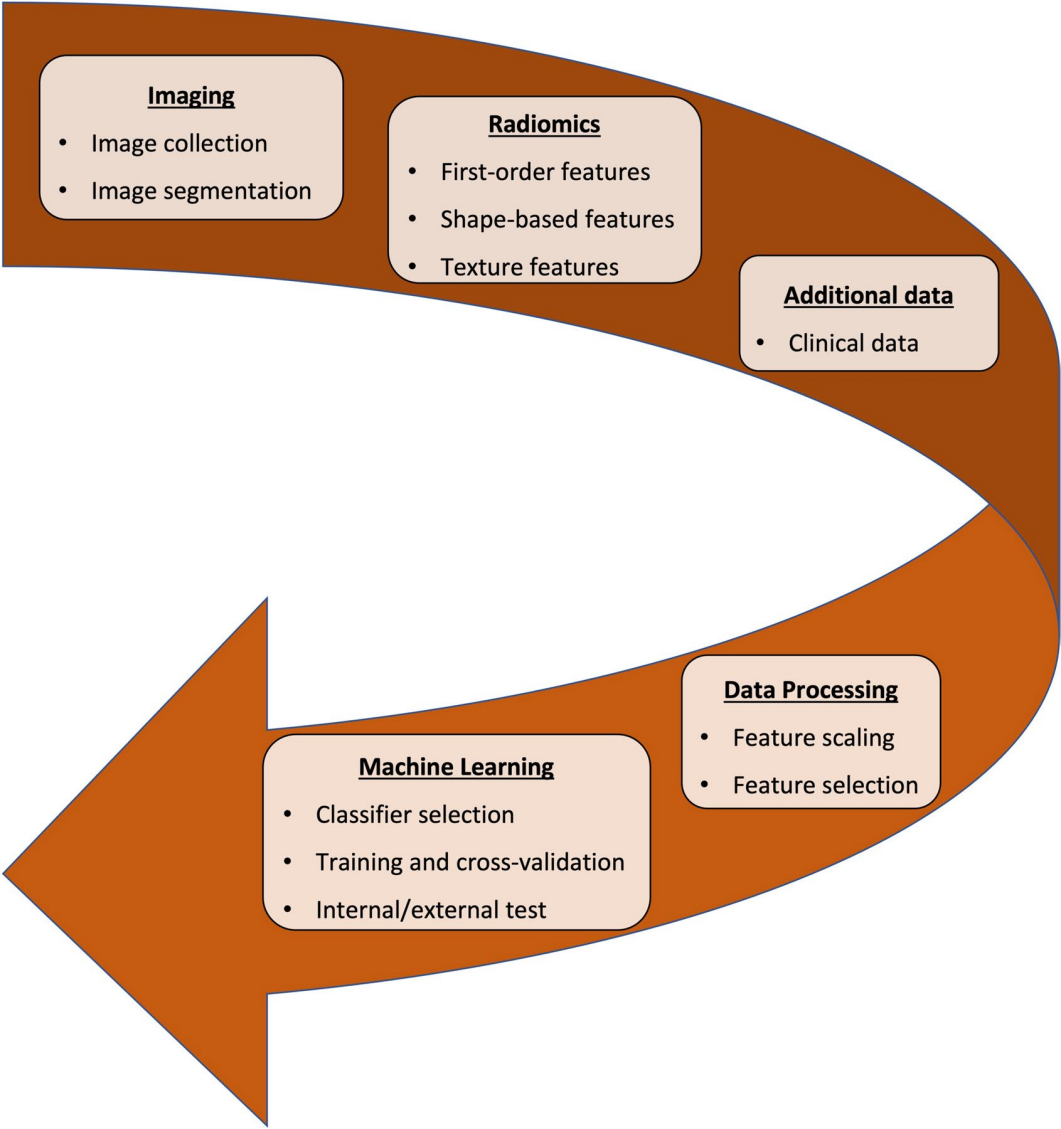
Example of machine learning radiomic workflow. A machine learning classifier can be employed to perform classification tasks based on radiomic features. Reproduced from: Fanciullo C et al. [69] [open-access article distributed under the terms of the Creative Commons Attribution License (CC BY)]

Radiomics involves a series of discrete steps, from image collection and segmentation to radiomic feature extraction and selection and, finally, classification model development, as summarized in Fig. 2. First, collected images are segmented using manual, semiautomated, or automated methods. In most studies, segmentation was manually performed by expert radiologists/clinicians or trainees under experts’ supervision [79]. Although the influence of interobserver variability on image segmentation can be evaluated as part of every radiomic workflow [80], semiautomated and automated approaches would ideally achieve higher reliability than manual segmentation. Second, several first-order, shape, and texture features are extracted and possibly combined with clinical information. Next, as most radiomic features are redundant and not informative [81], radiomic features are selected to create datasets which can be later mined. Finally, machine learning is used to perform classification tasks. Ideally, machine learning models are trained and validated using cross-validation on training datasets and then tested on independent datasets from different institutions. A clinical validation of the models against completely independent datasets is highly desirable to achieve clinical transferability. However, this independent or external validation is lacking in most radiomic studies dealing with musculoskeletal tumors [79], thus hampering generalizability of results. As musculoskeletal tumors are relatively rare entities, in particular sarcomas, free public repositories such as The Cancer Imaging Archive (https://www.cancerimagingarchive.net) may grant opportunities for research groups around the world to access data from different institutions and validate their models against independent datasets.

Deep learning can perform superior classification tasks compared to conventional machine learning. Particularly, deep learning models consist of neural network architectures which enable automated feature extraction (instead of manual extraction as in conventional machine learning), thus improving the efficiency of image analysis and providing assistance for nonexpert users [82]. However, deep learning models need to be trained using larger sets of labeled data compared to conventional machine learning, which is why their application to uncommon musculoskeletal tumors is still limited. First, images are preprocessed to obtain suitable quality annotated data and then split into training, validation, and test datasets with appropriate proportions. Second, the model is trained on the training dataset. Third, the model performance is evaluated on the test dataset [82]. In musculoskeletal oncology, most imaging-based deep learning models were developed using radiographs, CT, and MRI for diagnosis-related tasks, such as tumor classification—benign *versus* malignant discrimination [22, 83] or grading [84]—and segmentation [85]. In particular, deep learning showed similar and better accuracy compared to musculoskeletal fellowship-trained radiologists and radiology residents, respectively, in classifying primary bone tumors on radiographs [22]. In another study dealing with MRI of bone lesions, deep learning could differentiate benign from malignant tumors on a par with experts [83]. Deep learning was also used in combination with radiomics, for instance to differentiate lung from non-lung spine bone metastases on dynamic contrast-enhanced MRI [86] or benign from malignant sacral tumors on CT [87]. In addition to studies dealing with classification of lesions, deep learning was applied to musculoskeletal tumors like osteosarcoma for automated segmentation purposes [85]. Finally, a very few studies applied deep learning to musculoskeletal tumors for outcome-related tasks, such as recurrence prediction in giant cell tumor of the bone after curettage based on pre-operative MRI [88]. Overall, although promising results have been published, insufficient training data prevent most deep learning models from being implemented into clinical practice.

In conclusion, radiologists are asked to play a key role in moving AI—including both conventional machine learning and deep learning methods—and radiomics of bone and soft-tissue tumors from theory to clinical practice. The main limitations of current research studies, such as the relatively low number of patients and the lack of external/independent validation, need to be addressed in future investigations. Public repositories and institutional infrastructures for multi-center collaboration may allow to overcome these limitations and accelerate the process of clinical implementation.

## Orthopedic implants and implant-related complication

Musculoskeletal radiologists routinely evaluate orthopedic implants for appropriate positioning and potential complications. With the increasing number of orthopedic implant surgeries being performed [89], AI-aided postoperative image analysis has the potential to reduce workload, minimize fatigue-related errors, increase speed, and improve efficiency.

A theoretical AI pipeline for implant evaluation may include several steps, from body part identification to implant assessment [90], as follows.

First, the body part of interest, laterality and radiographic views are identified. Deep learning showed up to 100% accuracy in classifying anatomic region based on musculoskeletal radiographs [91]. Similarly, deep learning algorithms had almost perfect accuracy in determining laterality on radiographs [92] as well as radiograph position [93].

Second, the orthopedic implant is identified. Deep learning models demonstrated the ability to detect the presence of implants with up to 100% accuracy, including knee [32] and shoulder [33] arthroplasties, spinal hardware [31], and fracture fixation devices [93].

Third, the orthopedic implant is characterized into design types and models. The task of design typing includes differentiating between anatomic types of orthopedic implants. Particularly, deep learning models were developed to differentiate total from unicompartmental knee arthroplasty [32] as well as total from reverse total shoulder arthroplasty [33]. The task of identifying specific implant models is less relevant for post-operative radiological evaluation but crucial for revision surgery planning, as implant-specific tool kits are required. Orthopedic surgeons often spend time and efforts to identify implant models before revision surgery, for instance using orthopedic implant atlases of post-operative radiographs, with the risk of failure and potential negative impacts on outcome. A recent systematic review reported good to excellent performance of deep learning in classifying orthopedic implant models on radiographs [94], and one study demonstrated better performance of deep learning compared to non-deep learning AI algorithms [95].

Fourth, the orthopedic implant position is evaluated. Deep learning algorithms could measure inclination and version of the acetabular component after total hip arthroplasty, and little difference in measurements was found between human reader and AI [34]. Other orthopedic measurements such as lumbar lordosis [96] and lower limb length [97] could be obtained from radiographs automatically using AI, while saving time compared to manual calculations. Additionally, some AI algorithms for implant position assessment are already incorporated into commercially available orthopedic software [98].

Fifth, implant-related complications are identified. An important consideration is the relative rarity of complications after orthopedic implant surgery, which is (luckily) observed in clinical practice and results in unbalanced classes, thus limiting AI analysis. Deep learning models achieved 70% accuracy in detecting loosening after total knee or total hip arthroplasty on radiographs, which was improved when combining imaging and clinical information [35]. AI may potentially help predicting other implant-related complications, such as periprosthetic fractures, dislocation, periprosthetic infection, and component wear, which would be beyond human perception. AI-aided prediction of post-operative complications is in its early stage of development, and, given their tremendous implications for surgical outcome, it deserves future investigation.

Further research is also needed to compare AI alone and as an adjunct with human experts in evaluating orthopedic implants [99].

## Conclusion and future perspectives

This narrative review provided an overview of AI clinical applications in musculoskeletal imaging. Most studies evaluated the performance of AI algorithms compared to expert radiologists. Experts usually have very high accuracies, but many years of training and experience are required to achieve expert-level [100].

Thus, studies emulating real-life practice settings, including readers with different levels of expertise, are needed to fully understand the added value of AI in musculoskeletal diseases and bridge the gap between research and clinical application. It is important to mention that some AI technologies in musculoskeletal radiology are already commercially available, such as algorithms for fracture detection, bone age estimation, and osteoarthritis quantification [101].

As the number of AI products continues to increase, it will be crucial for radiologists to play a role in the selection and application of these technologies. Hence, rather than replacing radiologists, the use of AI may instead help them to optimize workflow, augment diagnostic performance, and keep up with ever-increasing workload. This also entails that legal liability is ultimately assigned to a human authority, namely the radiologist, who should take the responsibility [102].

## Data Availability

Data can be obtained upon request to the corresponding author.

## Abbreviations

AI: Artificial intelligence
AUC: Area under the curve
CT: Computed tomography
MRI: Magnetic resonance imaging
